# Laparoscopic anatomical liver resection after complex blunt liver trauma: a case report

**DOI:** 10.1186/s40792-018-0432-5

**Published:** 2018-03-23

**Authors:** Arpad Ivanecz, Vid Pivec, Bojan Ilijevec, Saša Rudolf, Stojan Potrč

**Affiliations:** 1 Department of Abdominal and General Surgery, University Medical Center Maribor, Ljubljanska ulica 5, 2000 Maribor, Slovenia; 2 Department of Radiology, University Medical Center Maribor, Ljubljanska ulica 5, 2000 Maribor, Slovenia

**Keywords:** Laparoscopic, Anatomical liver resection, Blunt liver trauma, Complications, Bile leak

## Abstract

**Background:**

Various minimally invasive therapies are important adjuncts to management of hepatic injuries. However, there is a certain subset of patients who will benefit from liver resection, but there are no reports in the literature on laparoscopic anatomical liver resection for the management of complications after blunt liver trauma.

**Case presentation:**

A 20-year-old male was admitted to the Emergency Unit of a tertiary referral center following a car accident. The patient was hemodynamically stable, and a radiologic workup demonstrated an isolated grade 3 injury of the left hemiliver. Initially, a nonoperative management was indicated, but during days following the injury, a high-volume biliary fistula complicated the clinical course. Despite percutaneous drainage, the development of devastating consequences of biliary peritonitis was imminent. A pure laparoscopic anatomical liver resection was performed. Left lateral sectionectomy eliminated the source of bile leak, and the surgery was completed with abdominal cavity lavage. Postoperative outcome was uneventful, and the patient was discharged on day 9 after injury and day 4 after surgery returning to his normal activity.

**Conclusions:**

In highly selected, hemodynamically stable patients with no other life-threatening concomitant injuries, laparoscopic liver resection in elective setting is feasible and safe for the management of complications after complex blunt trauma of the left liver. Extensive experience with hepatic surgery is needed, and surgeons should understand the increased risk they assume by taking on more complex surgical techniques.

## Background

Complex blunt liver trauma (BLT) still carries a high morbidity and mortality rate. Uncontrolled hemorrhage leading to exsanguination is the leading cause of death [1]. Another consequence of injury is a posttraumatic bile leak, and a high-volume biliary fistula can hamper patient’s recovery seriously [2].

Nonoperative management (NOM) of BLT has become the standard of care for hemodynamically stable patients. Minimally invasive therapies like angiography, image-guided percutaneous drainage, endoscopic retrograde cholangiopancreatography (ERCP), and laparoscopy remain important adjuncts to NOM of hepatic injuries [3].

Nevertheless, there is a certain subset of patients with BLT who will benefit from a resectional therapy. Liver resection (LR) of the injured portion of liver can definitively control bleeding, eliminate devitalized tissue, and avoid bile leak [4].

Laparoscopic LR has become a standardized surgical technique, but its role in BLT is not defined [5]. To the best of our knowledge, this is the first reported case of laparoscopic anatomical LR for the management of complications after BLT.

## Case presentation

### Sunday—day of the injury

A 20-year-old male was admitted to the Emergency Unit of a tertiary referral center following a car accident. The car crashed into a gutter on the local road. The exact mechanism of the injury was unknown; the patient was found unconscious sitting in the car, but hemodynamically stable and normopneic with 100% SpO2. Clinical examination showed bruises and upper abdomen tenderness. Acute alcohol poisoning and drug intoxication with cocaine and amphetamine were confirmed. Laboratory levels of hemoglobin (132 g/L) and hematocrit (0.38) were normal. Ultrasound (US) revealed the presence of free fluid diffusely in the abdominal cavity estimated to 700 ml. Intravenous contrast-enhanced computed tomography (CT) scan demonstrated a large (9 × 5 cm) liver parenchyma disruption dividing segments 2 and 3 from 4a and 4b at the level of falciform ligament. No source of active bleeding was revealed, and a diagnosis of isolated grade 3 BLT (laceration with parenchymal depth more than 3 cm) was confirmed. Liver injury was graded according to the organ injury scale drawn up by the American Association for the Surgery of Trauma [6]. The patient was transferred to intensive care unit (ICU) and remained stable; thus, NOM was indicated.

### Monday

After 1 day in the ICU, the patient continued to be hemodynamically stable with normal levels of hemoglobin (124 g/L) and hematocrit (0.37). A control abdominal US revealed no significant changes.

### Tuesday

**Fig. 1:**
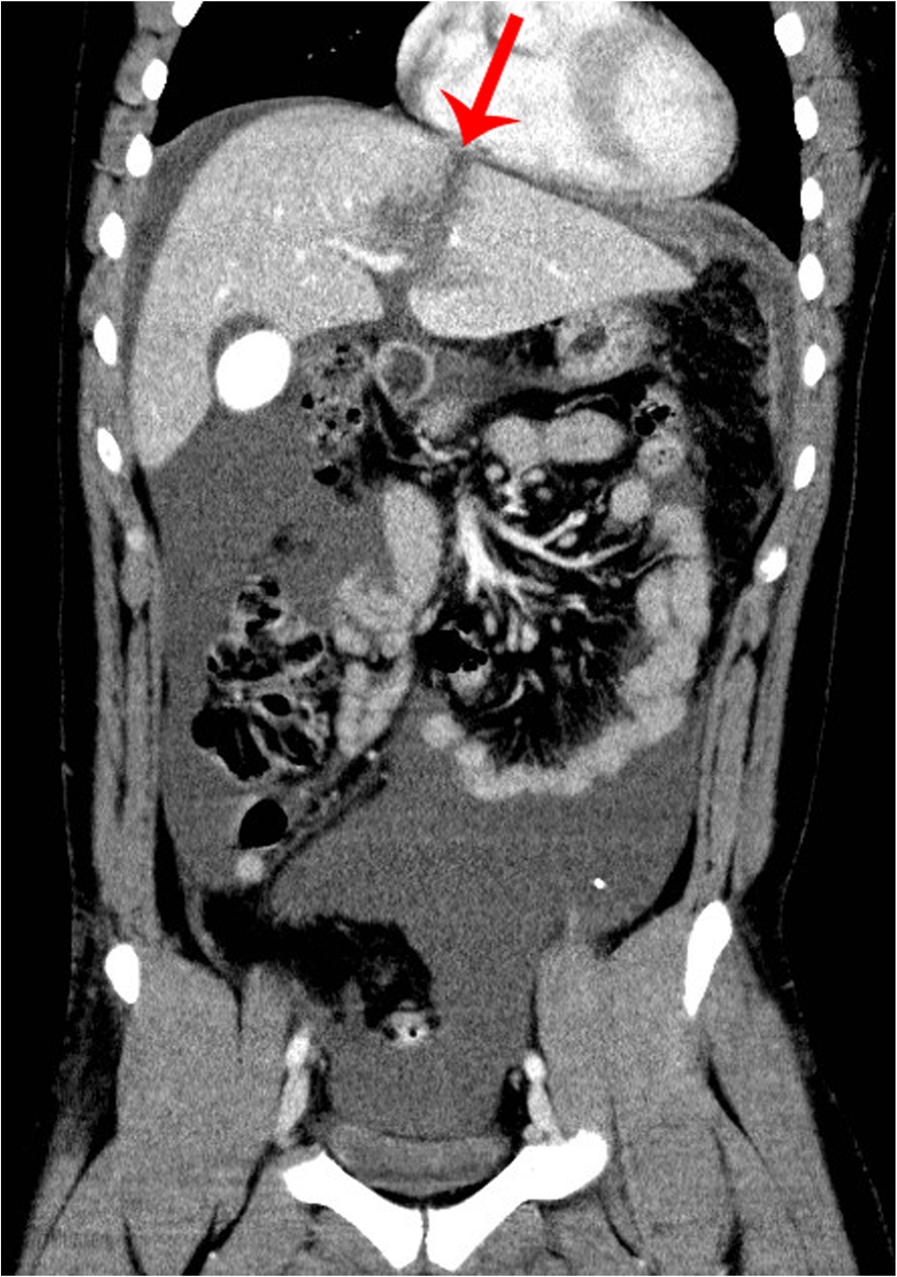
Following clinical deterioration, a repeated computed tomography (CT) scan demonstrated a localized grade 3 liver injury again and significant increase of free fluid in the abdominal cavity. The intended resection line was already outlined by traumatic rupture on the left side of the falciform ligament and indicated by arrow

On day 2, the patient started to complain of weakness, nausea, and abdominal pain and deteriorated. He developed fever, leucocytosis, and highly elevated C-reactive protein (177 mg/L), but remained hemodynamically stable with only slightly decrease in hemoglobin (112 g/L) and hematocrit (0.33) levels. A repeated CT scan demonstrated localized liver injury, and significant increase of free fluid in the abdominal cavity, again, no extravasation of contrast was seen (Fig. 1). The condition was diagnosed as peritoneal inflammatory reaction, and minimally invasive intervention was considered. US guided percutaneous drainage revealed presence of 2.5 L of bile mixed with some blood in the abdomen. A drain was placed into the recto-vesical pouch.

### Wednesday and Thursday

**Fig. 2:**
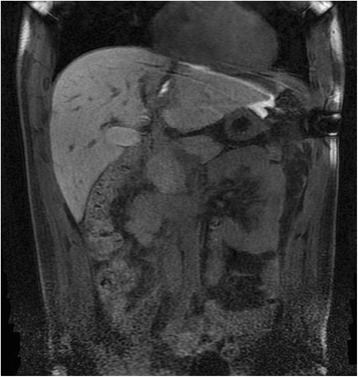
Magnetic resonance imaging (MRI) with hepatocyte-specific contrast agent showed a biliary leak originating from terminal branches of the left liver, with high signal of contrast collection under the left diaphragm

On days 3 and 4, the patient recovered well; however, the drain output was rather high: 700 mL of bile/day with extremely elevated level of bilirubin in drain content (1262 μmol/L). Magnetic resonance imaging (MRI) with hepatocyte-specific contrast agent Gd-EOB-DTPA (Primovist®, Bayer Schering) showed a biliary leak originating from terminal branches of the left liver, with high signal of contrast collection under the left diaphragm (Fig. 2).

### Friday

Elective surgical intervention has been considered. Laparoscopic anatomical LR was planned; according to the MRI, a left lateral sectionectomy (LLS) was indicated. Patient was positioned in standard supine position with the operating surgeon standing on the patient’s right and one assistant on the opposite side. A four-trocar configuration was used (three 12-mm trocars and one 5-mm trocar), positioned in a rhomboid configuration in the upper abdomen. A continuous carbon dioxide pneumoperitoneum was induced to a pressure of approximately 12 mmHg. Laparoscopic exploration revealed an intense biliary peritonitis. The bile was aspirated from the entire abdomen, and lavage was performed.

**Fig. 3:**
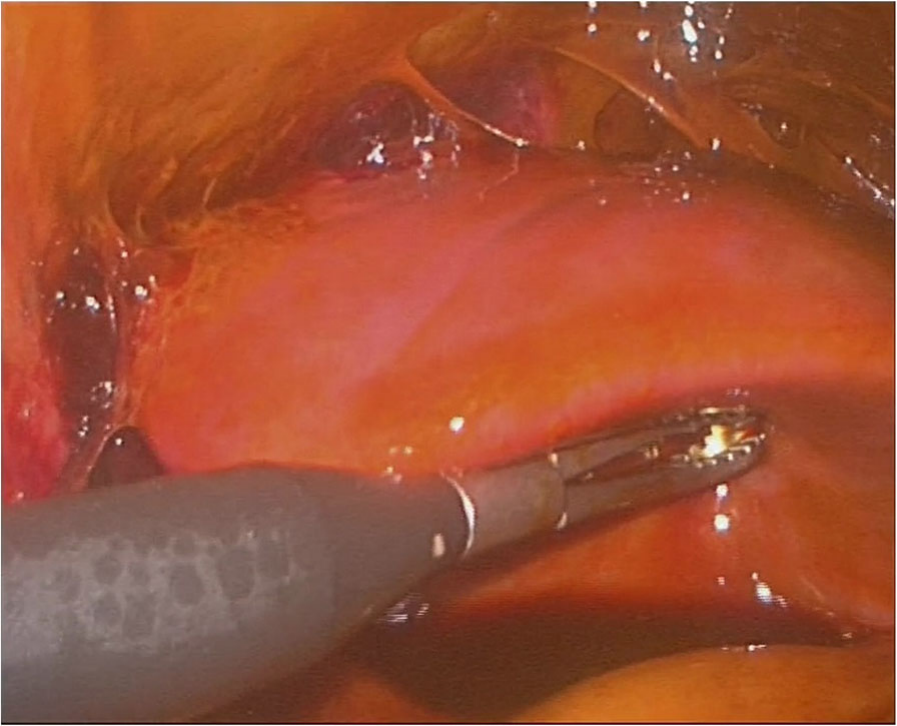
The intended resection line was already outlined by traumatic rupture on the left side of the falciform ligament. Note the bile collections

**Fig. 4:**
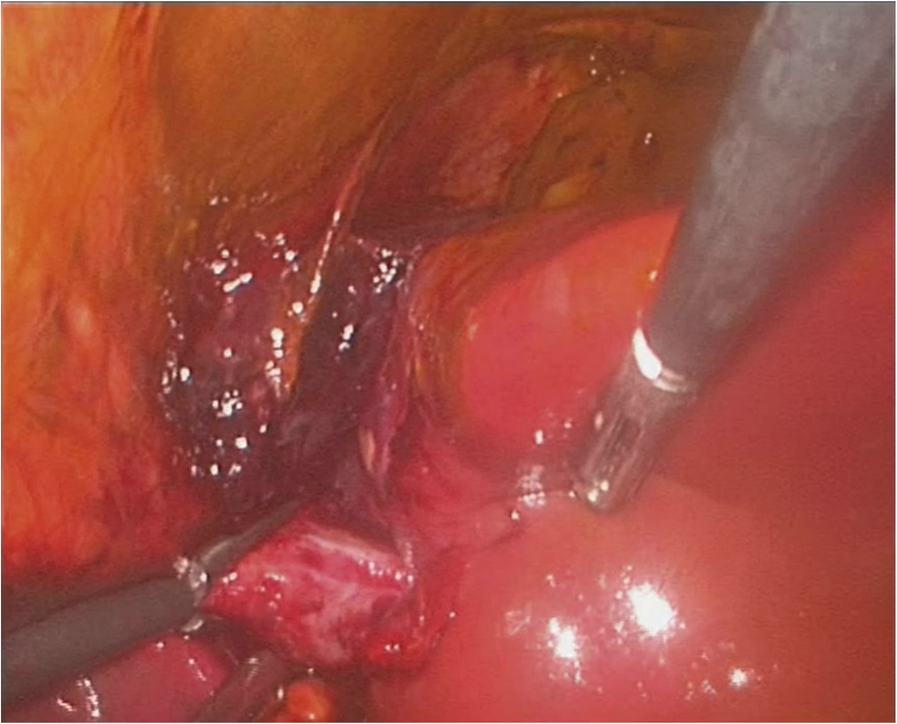
The rupture of the liver parenchyma caused by trauma complies with the transection line usually applied in formal left lateral sectionectomy (LLS) for any other indication. The portobiliary pedicles for segments 2 and 3 were easily defined

**Fig. 5:**
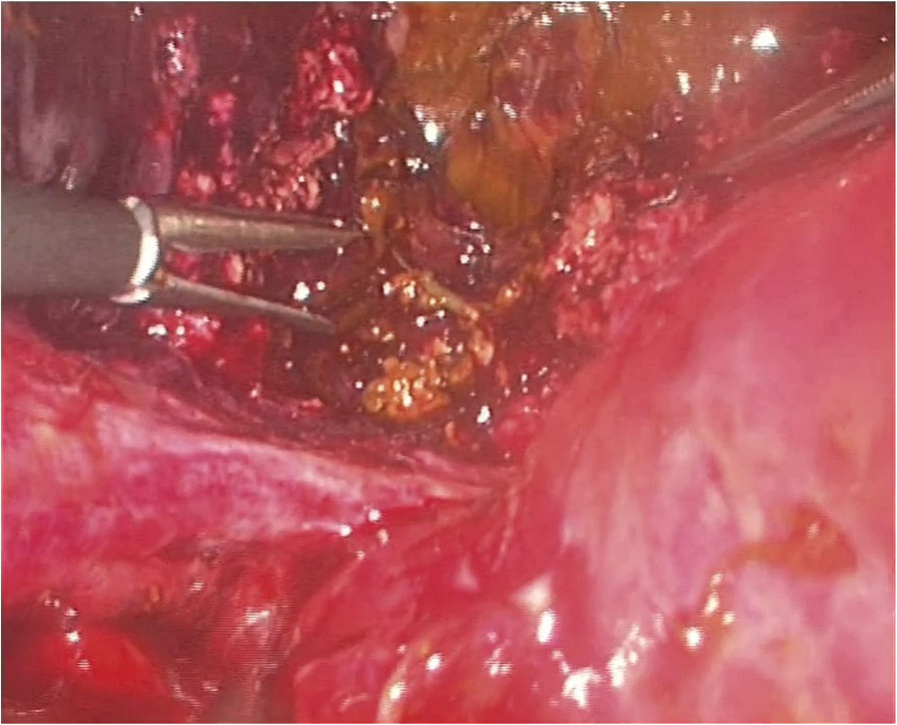
After aspirating the bile and blood clots and completing the transection plane by vessel-sealing device, the source of bile leak was clearly seen under direct vision

**Fig. 6:**
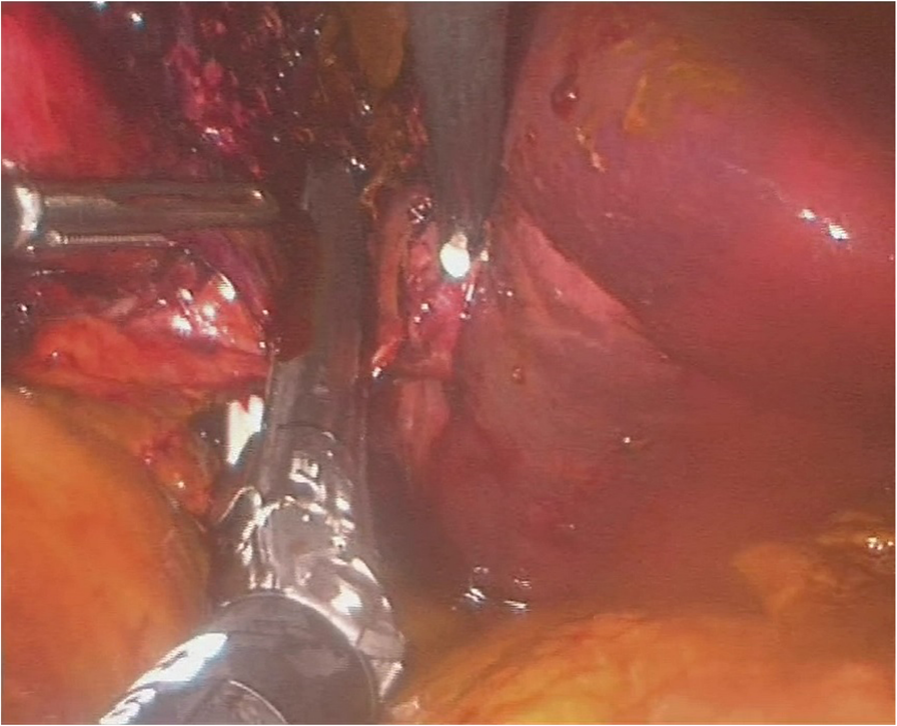
Completion of glissonian plane transection and gaining an inflow control

The liver was examined by direct vision and laparoscopic US. It was confirmed, that the intended resection line was already outlined by traumatic rupture on the left side of the falciform ligament (Fig. 3). There were no signs of active bleeding. After aspirating the bile and blood clots and completing the transection plane by vessel-sealing device LigaSure® (Covidien, Mansfield, MA), the portobiliary pedicles for segments 2 and 3 were easily defined (Fig. 4). The source of bile leak was clearly seen under direct vision: the biliary pedicles for the left lateral section of the liver were transected by trauma (Fig. 5). The portobiliary pedicles were dissected free and then transected under direct vision with an articulated linear stapler. Two cartridges were needed for completion of glissonian plane transection and gaining an inflow control (Fig. 6). After further parenchymal transection by vessel-sealing device, the hepatic resection was completed by transection of the left hepatic vein by third stapler. Left triangular and coronary ligaments were finally divided. The estimated blood loss was < 50 ml. A Pringle tap was positioned at the beginning of the procedure, but it was not required.

The resection margin was examined carefully for bleeding and bile leaks, and for this purpose, the pneumoperitoneum pressure was lowered. A bile leak was identified in the segment 4b and controlled by laparoscopic suturing with prolene 4/0 stitches. Repeated examination under irrigation and by gently pressing a small gauze swab against resected surface revealed no further bile leak. TachoSil® (Takeda Austria, GmbH, Linz, Austria) fibrin sealant patch was applied to the cut liver surface. The resected specimen was placed in the retrieval bag and removed without fragmentation through a separate 4–5-cm suprapubic Pfannenstiel incision. The rectus muscles were separated by lateral retraction and were not transected.

### Days after surgery

Postoperative outcome was uneventful, and the patient was discharged on the day 9 after injury and day 4 after surgery. Two weeks after injury, he returned to his normal activity.

## Discussion

The presented patient developed posttraumatic biliary leak after suffering complex-isolated BLT. Multidisciplinary management revealed a high-output biliary fistula. Following a failed NOM, an early surgical therapy was indicated. After laparoscopic anatomical LR, he recovered well and was discharged from hospital soon.

There are many important issues to be emphasized in this case. Firstly, the failed NOM of BLT is well described in the literature and associated with significant morbidity correlating with the grade of liver injury. In series of Kozar et al., 14% of patients developed hepatic complications of which 5% were having grade 3 injuries, 22% were having grade 4 injuries, and 52% were inpatients having grade 5 injuries, respectively [7]. Biliary leaks complicate liver trauma with a frequency of 0.5 to 21% [2, 8]. The management of high-volume biliary fistula poses a formidable challenge. Two days following the injury, the clinical presentation of this patient was suggestive of complications. CT confirmed the increase of the abdominal free fluid. As first line therapy, the simplest minimally invasive procedure was chosen and the collection was eliminated by US-guided percutaneous drainage. After noticing bile in the percutaneous drainage device, bile duct injury was highly suspicious. The diagnosis of major posttraumatic bile leak was confirmed by MRI, revealing a definite contrast leaking from the terminal branches of the left bile duct.

Another issue of this report is to outline that morbidity in high-grade liver trauma does not necessarily end with intervention. During days following the injury, a high-output biliary fistula has been recognized. Practically, the total amount of bile produced in a day (700 mL) was drained out of the body. However, after image-guided percutaneous drainage, the clinical condition improved and the patient became asymptomatic. In addition, MRI revealed only minimal free fluid in the abdomen, demonstrating an adequately functioning draining device. Nevertheless, the only issue of concern was the position of this drain in the recto-vesical pouch, thus permitting the bile to contaminate the entire abdomen before being eliminated from there. The development of devastating consequences of biliary peritonitis was imminent, which typically occurs days following the injury and can initially be clinically silent. Evidently, further management was required.

A special point of interest of this case concerns the choice between ERCP and early surgery. ERCP with ampullary sphincterotomy and biliary stent insertion was reported to be highly successful in controlling biliary leak. However, in the large series of Anand et al., the mean hospital length of stay after ERCP therapy was 33 days ± 21 days and the closure of the fistula was obtained not earlier than 47 days on average [8]. The requirement to repeat the ERCP procedures with stent exchange before the definite fistula closure is reported in the literature [2, 8]. Essentially, neither the optimal duration of leak before ERCP nor the duration of leak before operative consideration has been studied in the literature [3]. In this case, the bile contaminated the entire abdominal cavity; thus, it has been found to be necessary to perform at least a lavage and an additional drainage to prevent the development of devastating systemic consequences of biliary peritonitis. Although laparotomy remains an option, lavage and drainage can be safely and effectively performed by laparoscopy [9, 10]. Laparoscopic lavage in combination with trans-cystic biliary drainage and application of surgical tissue sealing patch on the bile leak has been reported in the literature; however, that patient was hospitalized for as long as 18 days after surgery [10]. Actually, the therapeutic decision in our case has been based on the elimination of the bile source and accomplished by laparoscopic anatomical resection of the injured left lateral section of the liver. A bile leak from the segment 4b was controlled by laparoscopic suturing. Importantly, no additional invasive therapy was required and the benefit of surgery was evident by the patient’s early recovery in the absence of any complications.

The significant value of this report is to present the feasibility and safety of the laparoscopic anatomical LR for the management of complications after BLT. Extensive literature search on laparoscopic LR was made, and it was based on recently published systematic review of over 9000 cases reported by 179 single centers [11]. Laparoscopic débridement of the liver abscess after BLT have been described previously; however, it was a removal of necrotic tissue only and not a formal anatomical hepatic resection [12]. To the best of our knowledge, this case report is the first description of laparoscopic anatomical LR after BLT. Notwithstanding, the authors would like to emphasize that laparoscopic anatomical LR is a viable option but only in appropriate circumstances and with adequate expertise. Basically, the rupture of the liver parenchyma caused by trauma in this patient complies with the transection line usually applied in formal LLS for any other indication. Laparoscopic LLS is reported to be a feasible, safe, and efficient procedure, associated with a quick, smooth learning curve [13]. Importantly, the patient was continuously hemodynamically stable; moreover, blood transfusions were not required at any moment and the surgeons and staff were comfortable with the proposed procedure. It should be recognized that the surgical procedure was performed in an elective setting with dedicated laparoscopic team with expertise and extensive experience in hepatic surgery. Furthermore, there is no doubt that the absence of concomitant injuries facilitates patient’s uneventful and early recovery. However, by laparoscopic approach, an extensive abdominal incision has been avoided. Four 5–12-mm port site and a small suprapubic Pfannenstiel incisions represented only a minimal injury to the abdominal wall. The almost intact integrity of the musculature permitted patient’s early returning to his normal activity. The better cosmetic effect in comparison with laparotomy, particularly in this young man, should not be neglected as well.

## Conclusions

Laparoscopy has gained a role as diagnostic and therapeutic procedure in the treatment of complications following NOM of BLT. There are several other adjunctive therapies available; however, a subset of patients might benefit from a definitive resectional therapy. In this case, the resection of the injured portion of the left liver eliminated the devitalized tissue and controlled the bile leak. The value of this report is to present the feasibility and safety of the laparoscopic anatomical LR for the management of complications after BLT of the left hemiliver, enhancing an early recovery of patient.

## Abbreviations

BLT: Blunt liver trauma
CT: Computed tomography
ERCP: Endoscopic retrograde cholangiopancreatography
Gd-EOB-DTPA: Gadolinium ethoxybenzyl diethylenetriamine pentaacetic acid
ICU: Intensive care unit
LLS: Left lateral sectionectomy
LR: Liver resection
MRI: Magnetic resonance imaging
NOM: Nonoperative management
US: Ultrasound
